# Management of hypertension addressing hyperuricaemia: introduction of nano-based approaches

**DOI:** 10.1080/07853890.2024.2352022

**Published:** 2024-05-16

**Authors:** Koyeli Girigoswami, Radhakrishnan Arunkumar, Agnishwar Girigoswami

**Affiliations:** a Medical Bionanotechnology, Faculty of Allied Health Sciences, Chettinad Hospital & Research Institute (CHRI), Chettinad Academy of Research and Education (CARE), Chennai, India; b Department of Pharmacology, Chettinad Hospital & Research Institute (CHRI), Chettinad Academy of Research and Education (CARE), Chennai, India

**Keywords:** Xanthine oxidase, febuxostat, allopurinol, blood pressure, uric acid

## Abstract

Uric acid (UA) levels in blood serum have been associated with hypertension, indicating a potential causal relationship between high serum UA levels and the progression of hypertension. Therefore, the reduction of serum UA level is considered a potential strategy for lowering and mitigating blood pressure. If an individual is at risk of developing or already manifesting elevated blood pressure, this intervention could be an integral part of a comprehensive treatment plan. By addressing hyperuricaemia, practitioners may subsidize the optimization of blood pressure regulation, which illustrates the importance of addressing UA levels as a valuable strategy within the broader context of hypertension management. In this analysis, we outlined the operational principles of effective xanthine oxidase inhibitors for the treatment of hyperuricaemia and hypertension, along with an exploration of the contribution of nanotechnology to this field.

## 1. Introduction

Since the kidneys perform vital functions, such as urine production and maintaining homeostasis, drug targeting to the kidneys remains a major research focus. A range of severe complications can result from kidney dysfunction [1]. The most common of these are elevated blood pressure (hypertension), inflammatory responses, susceptibility to urinary tract infections, formation of renal calculi (kidney stones) and a variety of other related issues [2,3]. Maintaining overall health requires understanding and addressing these potential complications owing to the intricate nature of renal health. Recent decades have seen a significant increase in research directed towards nephrolithiasis, a particularly serious condition [4]. In addition, this disease is associated with increased serum uric acid (UA) levels, enhanced urinary UA excretion and persistently low urine pH levels (<5.5). All of these factors contribute to the accumulation of UA calculi in the kidney pelvis, collecting ducts and ureters and cause pathological alterations in the kidneys [5–7].

UA is the ultimate by-product of the breakdown of purine. An imbalance involving excessive UA production, diminished excretion or amalgamation of both factors can lead to hyperuricaemia, which is distinguished by elevated UA levels [8,9]. Gout is caused by excessive UA production, diminished excretion or a combination of both. In addition to its association with gout, hyperuricaemia has been conclusively linked to a variety of other medical conditions, including hypertension and renal disease [10,11]. In addition to highlighting the systemic implications of elevated UA levels, this intricate interplay also indicates their relevance to broader health disorders beyond the confines of gout.

A frequently employed therapeutic approach for the supervision of hyperuricaemia in individuals suffering from gout involves the administration of allopurinol and febuxostat, which function as xanthine oxidase (XO) inhibitors [12,13]. By interfering with the conversion of hypoxanthine (HPX) and xanthine (XAN) to UA, they are effective in reducing urate synthesis, which is the basis for this therapeutic choice. Febuxostat is believed to exhibit a more robust hypouricemic effect than allopurinol, and a reduced risk of hypersensitivity syndrome [14]. Moreover, febuxostat has been demonstrated to maintain its safety and efficacy profile without requiring dose adjustments in patients with mild-to-moderate renal impairment. In patients with gout requiring careful consideration of renal function during treatment, this attribute enhances the therapeutic utility of febuxostat, providing clinicians with a valuable option for managing hyperuricaemia [15]. Both allopurinol and febuxostat have demonstrated effectiveness in reducing blood pressure and decelerating the progression of chronic kidney disease, as indicated by several studies. These medications exhibit dual benefits, extending beyond their primary roles in hyperuricaemia and gout management. It is believed that the mechanism of action of these drugs is a combination of lowering serum levels of UA, enhancing endothelial function, mitigating oxidative stress, preventing the development of glomerular hypertension, thickening of afferent arterioles and histological changes indicative of ischemic renal damage [16,17].

The lower solubility and reduced half-life of a drug via the oral route result in reduced bioavailability, underscoring the significance of developing sustained-release formulations, particularly for XO inhibitors such as allopurinol [18]. The use of colloidal drug carriers for enhancing bioavailability is becoming increasingly popular, and nanoparticles are emerging as promising candidates [19–21]. In nanotechnology, therapeutic agents are encapsulated, entrapped or adsorbed onto the surfaces of micelles, metal nanoparticles, solid polymeric colloidal particles or vesicular structures [22–26]. A range of natural and synthetic materials can be used to make these nanoparticles, including chitosan, alginate and gelatin, with nanometre size [27,28]. Several factors must be considered when choosing an ideal material for nanoformulation development. The key considerations here are the desired size of the nanoparticles as well as the desired drug release profile of the nanoparticles. The selection of nanomaterials must also consider factors such as biocompatibility, toxicity, biodegradability, inherent physicochemical properties of the drug and surface characteristics of the formulation [29,30]. This review discusses the chemistry and mechanism of XO inhibitors, specifically allopurinol and febuxostat, in the treatment of gout and their potential impact on hypertension. Simultaneously, this review explores research efforts focused on nanoformulated allopurinol and febuxostat to enhance their solubility and bioavailability.

## 2. Working principle of XO

XO, also known as XAN oxidoreductase, plays a key role in the catabolism or breakdown of purine nucleic acids in many species including humans. Its primary function is to catalyse the conversion of purines such as guanine monophosphate and adenosine monophosphate (AMP) into HPX or XAN [31]. HPX and XAN are further broken down into UA by XO. Uricase transforms UA into allantoin, a water-soluble molecule excreted in the urine [32]. The significance of XO lies in its essential role in mammalian cells, where constant cell turnover results in continuous degradation and renewal of endogenous or ingested purines. The alternative form of XO is xanthine dehydrogenase (XD), which is interchangeable with XO and catalyses the same reactions [33]. Although they utilize different cofactors to perform the same reaction, they differ in the way oxygen is utilized as a substrate in XO, while reduced form of Nicotinamide Adenine Dinucleotide (NADH) is utilized in XD [34]. XO is a homodimer with a reported molecular weight (MW) of 283–290 kDa and consists of two catalytically independent subunits, each of which has a mass of 150–155 kDa [35]. Monomers contain three subunits, each of which has a mass of 20, 40 or 85 kDa. The separation of these subunits is possible only under robust denaturing conditions. Within each monomer, distinct components contribute to the enzyme structure and function. Specifically, the 20 kDa subunit accommodates two iron–sulphur clusters (Fe2–S2), adding a crucial element to its composition [36]. In the 40 kDa subunit, a flavin adenine dinucleotide (FAD) molecule plays a significant role in enzymatic processes. Furthermore, the 85 kDa subunit hosts the molybdenum cofactor (Mo-co), which is intricately bound to the enzyme as molybdopterin [37]. This multicomponent structure highlights the complexity and precision involved in the functioning of the enzyme, with each constituent playing a distinct and essential role in its overall activity. Although each complete monomer with a MW of 150 kDa, demonstrated catalytic activity, the smaller fragments lacked the capability to catalyse the XO reaction. This deficiency arises from the indispensability of all cofactors in catalytic processes. In other words, the absence of any cofactors in the smaller fragments prevents them from exhibiting the enzymatic activity required for the XO reaction [38].

**Figure 1.**
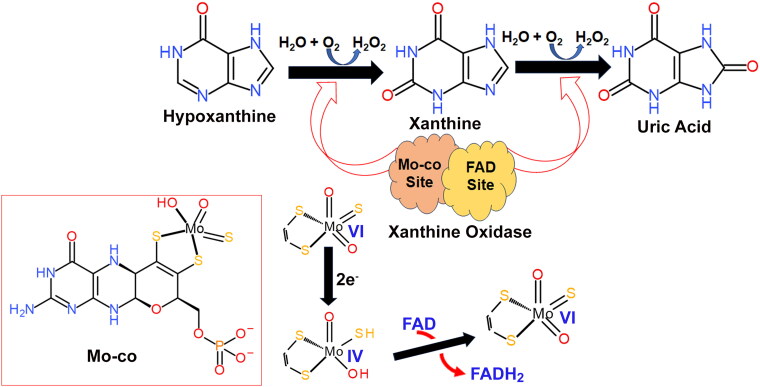
Role and mechanism of XO to convert HPX to UA.

From a structural standpoint, the XO homodimer enzyme has a subunit comprising 1333 amino acid residues [39]. XO serves as a molybdenum (Mo)-dependent enzyme responsible for catalysing two pivotal steps that limit the breakdown rate of purine nucleotides [40]. XO is a key enzyme in biochemical processes, specifically in the conversion of HPX to XAN [41]. Following this initial step, XO continues its catalytic function by further transforming XAN into UA. Notably, the successful conversion of XAN to UA by XO relies on the presence of a Mo-co, emphasizing the essential role of this cofactor in the enzymatic activity of XO [42]. The intricate enzymatic pathway involving XO is significant in the metabolism of purine compounds, ultimately contributing to the production of UA in biological systems. This intricate biochemical process unfolds through a series of four distinct reaction steps. Initially, a proton was transferred from the hydroxyl group of the Mo-co to Glu1261, a key event that activates the hydroxyl group [43]. The activated hydroxyl group then participates in a nucleophilic attack on XAN, marking the commencement of the catalytic process. Subsequently, in the second step, a hydride is relocated from the tetrahedral transition compound to the sulphur atom of the Mo cofactor. This transfer leads to the reduction of Mo from its higher oxidation state of Mo(VI) to Mo(IV), a crucial reaction in the enzymatic cycle [38]. In the third step, UA is generated through the protonation of an intermediate by Arg880. This step represents a pivotal transformation in the pathway and contributes to the ultimate formation of UA. As illustrated in Figure 1, the completion of these steps is associated with the reduction of FAD to FADH2. Simultaneously, this process oxidized Mo back to the initial oxidation state of Mo(VI). This coordinated redox activity highlights the intricate interplay between various cofactors and substrates within the XO enzymatic mechanism. It is noteworthy that, despite the recognition of XO as a source of superoxide, it has traditionally not been considered the primary contributor to reactive oxygen species (ROS) in cellular processes [44]. XO has been extensively implicated in many diseases including gout, UA stone formation and xanthinuria. As a result of the heightened activity of XO, the UA level increases, causing conditions such as gout and hyperuricaemia. Correspondingly, UA precipitates in joints, skin and blood capillaries, forming needle-like crystals that cause pain. The formation of kidney stones is also related to UA, which can form sodium urate crystals [45]. In contrast, xanthinuria is a unique genetic disorder that is associated with low XO activity. Consequently, elevated levels of circulating XAN can occur as a result of this disorder, which is associated with several diseases, including renal failure.

## 3. Health hazards related to UA level

Males have a higher serum UA level than females in the general population. The hypothesis is that oestrogen and progesterone, which are produced by females, may lower UA levels, contributing to this sex difference. Additionally, studies have reported that the impact of genetic variants on hyperuricaemia is more significant than that of dietary exposure in the common population. Overproduction of UA along the purine breakdown pathway, which primarily occurs in the liver, is one of the causes of hyperuricaemia. An additional factor in the development of hyperuricaemia is insufficient elimination of UA by the kidneys, involving disruptions in reuptake carriers [46]. Key players in this process include urate transporter 1 (URAT1), glucose transporter 9 (GLUT9) and secretory transporter ATP-binding cassette subfamily G member 2 (ABCG2) [47]. The malfunction of URAT1 and GLUT9, responsible for the reuptake of UA, can lead to a breakdown in the regulation of UA levels within the kidneys. Similarly, impairment of the secretory transporter ABCG2 can result in inadequate removal of UA from renal tubules, contributing to the accumulation of UA in the bloodstream. Generally speaking, hyperuricaemia is defined as a blood UA level exceeding 7 mg/dL, as the formation of UA crystals starts when the blood UA level reaches 6.8 mg/dL at 37 °C, and therefore, hyperuricaemia is considered a significant risk factor for conditions like gout and urolithiasis [48]. Metabolic imbalances in obesity, insulin resistance, dyslipidaemia and hypertension are intimately linked to hyperuricaemia, gout and urolithiasis. As a result, elevated UA levels are often considered as surrogate markers of metabolic syndrome. According to the homeostasis model assessment of insulin resistance, UA levels are linearly correlated with fasting insulin levels, and a linear increase in UA levels correlates with a linear increase in insulin resistance measured using the homeostasis model assessment of insulin resistance [49]. It is interesting to note that fasting glucose and haemoglobin A1c levels are related to UA levels in a bell-shaped manner. In addition, in patients with type-1 diabetes mellitus, glycosuria has been identified as an important mechanism by which urinary UA is excreted, and it has been found that sodium-glucose cotransporter 2 (SGLT2) inhibitors can facilitate glycosuria and urate excretion [50]. A mechanism or procedure for the reduction of UA levels in hyperglycaemia involves glycosuria-induced UA secretion through GLUT9 isoform 2 or similar transporters at the proximal tubule, as well as GLUT9 isoform 2 inhibiting the uptake of UA from the collecting duct of the renal tubule, causing a reduction in UA levels. However, evidence suggests that gout can protect against diseases related to the central nervous system. In addition to Alzheimer’s disease, Parkinson’s disease, vascular dementia and non-vascular dementia, this protective effect extends to these conditions.

## 4. Treatment for higher UA level: XO inhibitors

### 4.1. Allopurinol

**Figure 2.**
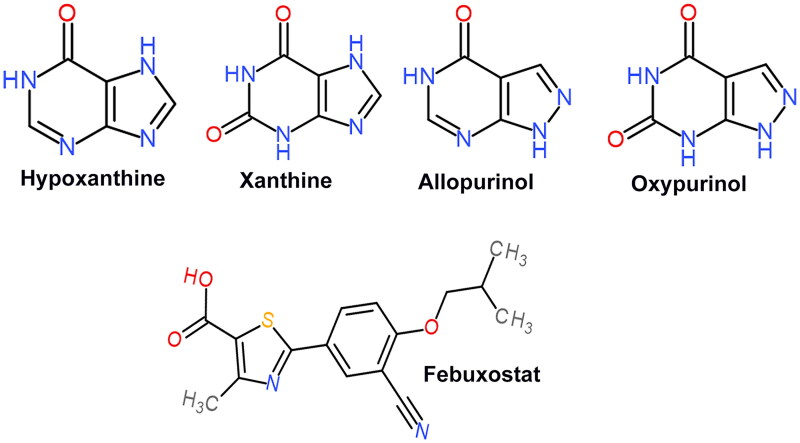
Chemical structures of HPX, XAN, allopurinol, oxypurinol and febuxostat.

Allopurinol, a drug that resembles HPX, has been used for over 50 years as a treatment for gout and has been widely used to reduce UA levels in the bloodstream [51]. Allopurinol hinders the activity of XAN oxidoreductase, the enzyme responsible for overseeing the last two phases of purine breakdown: the oxidative conversion of HPX to XAN, and the consecutive transformation of XAN into UA. XO transforms allopurinol into its primary metabolite, oxypurinol. Allopurinol and oxypurinol share structural similarities with the purine bases HPX and XAN, respectively (Figure 2). They competitively attach to XO, consequently impeding the enzyme’s role in converting HPX to XAN and subsequently XAN to UA. The administration of allopurinol therapy results in elevated levels of HPX and XAN in both the urine and plasma, accompanied by a reduction in UA concentrations in the plasma [52]. Allopurinol also acts by reducing de novo purine biosynthesis, a process believed to result from the feedback inhibition of amidophosphoribosyl transferase, an enzyme that governs the rate-limiting step in purine biosynthesis. At low concentrations, allopurinol serves as both a substrate for the enzyme and a competitive inhibitor. Conversely, at higher concentrations, it assumes the role of a non-competitive inhibitor. Oxypurinol is a non-competitive inhibitor of this enzyme [53]. The creation of this compound, coupled with its prolonged retention in tissues, accounts for a significant portion of the pharmacological activity associated with allopurinol.

In pharmacokinetics, allopurinol exhibits swift absorption, with peak plasma concentrations achieved within a relatively short timeframe of 30–60 min following oral administration [54]. This rapid absorption highlights the efficiency of drug uptake into the bloodstream, influencing its onset of action and therapeutic effects. Oxypurinol is characterized by lower oral bioavailability than allopurinol. Allopurinol has a relatively short half-life in the plasma, ranging from 2 to 3 h. In contrast, the half-life of oxypurinol is significantly prolonged, ranging from 14 to 30 h [55]. This extended half-life is attributed to renal reabsorption, which contributes to the sustained presence of oxypurinol in the blood stream. Allopurinol hinders XAN oxidoreductase activity via two distinct mechanisms. Initially, the primary drug allopurinol served as a substrate for XAN oxidoreductase. However, the resulting complex between the reduced and oxidized products, oxypurinol, is exceptionally stable. The breakdown of the complex occurs at a notably slow rate, although it occurs somewhat more rapidly in the presence of XAN, resulting in the formation of oxypurinol [56]. The interaction between allopurinol and XAN oxidoreductase is pseudo-irreversible. Another method of inhibiting XAN oxidoreductase involves oxypurinol, even in the absence of allopurinol. Oxypurinol, which is potentially generated through the action of aldehyde oxidoreductase, demonstrates a strong affinity for the reduced form of XAN oxidoreductase, effectively inhibiting the enzyme. This pathway is the second-most acceptable mechanism of inhibition.

The metabolic breakdown of allopurinol is a multifaceted process characterized by extensive transformation. This complicated series of metabolic reactions involves two primary pathways. Firstly, allopurinol undergoes XO-mediated conversion, leading to the formation of its active counterpart, oxypurinol, which serves as an inhibitor of the enzyme [57]. Secondly, the compound undergoes further modification through the action of phosphoribosyltransferases, specifically hypoxanthine-guanine phosphoribosyltransferase and orotate phosphoribosyltransferase. At this stage, allopurinol gives rise to nucleotide analogs, adding another layer of complexity to its metabolic fate [58]. Predictably, owing to their structural resemblance to natural purines and pyrimidines, not only do allopurinol and its derivative oxypurinol exhibit inhibitory effects on XO; however, they also extend their influence to other enzymes within the purine and pyrimidine metabolic pathways. This includes the inhibition of purine nucleoside phosphorylase and orotidine-5′-monophosphate decarboxylase [59]. Although allopurinol is generally recognized for its satisfactory efficacy and safety profile, it is essential to acknowledge the occurrence of exceedingly rare and severe adverse reactions associated with its administration. These adverse effects include interstitial nephritis, kidney or renal failure, liver impairment, vasculitis and a spectrum of skin rashes ranging from mild to highly severe, potentially leading to life-threatening conditions. The higher incidence of severe adverse reactions to allopurinol in patients with renal insufficiency may be due to the accumulation and metabolic effects of allopurinol, oxypurinol and their derivatives beyond XO inhibition [60]. This is underscored by their primary renal excretion.

Evidence from both randomized controlled trials (RCTs) and observational studies by van der Pol et al. suggested that allopurinol treatment can lower cardiovascular risk in individuals with hyperuricaemia [61]. However, the quality of evidence from RCTs is currently assessed as being low to moderate. Gois and Souza highlighted that existing RCT data are insufficient to ascertain whether UA-lowering therapy effectively reduces blood pressure, and they emphasized the necessity for additional studies to address this question definitively [62]. To conclusively determine whether allopurinol effectively reduces the risk of cardiovascular events, a meticulously designed and sufficiently powered randomized placebo-controlled trial is essential, particularly in high-risk patients with hyperuricaemia. In their clinical trial, Badve et al. did not observe greater effectiveness of allopurinol compared to placebo in decelerating the decline in estimated glomerular filtration rate over a 104-week period among patients with stage 3 or 4 chronic kidney disease and an increased risk of disease progression [63]. A study by Mackenzie et al. revealed that the use of allopurinol at a daily dosage of 600 mg did not show any enhancement in cardiovascular outcomes when compared with standard care among patients with ischemic heart disease [64]. Consequently, based on the ALL-HEART study results, it is not advisable to recommend allopurinol for secondary prevention of cardiovascular events in individuals with ischaemic heart disease.

### 4.2. Febuxostat

Febuxostat serves as a non-purine inhibitor of XO and plays a role in purine catabolism. Febuxostat differs structurally from allopurinol because it lacks a purine ring (Figure 2). Febuxostat belongs to the category of thiazole-carboxylic acid derivatives and exhibits distinctive selectivity in its mechanism of action [65]. It effectively inhibits both the oxidized and reduced forms of XO, which is a crucial enzyme involved in purine metabolism. It is a more selective and potent XO inhibitor than allopurinol. The structural dissimilarity between febuxostat and purine or pyrimidine compounds makes it a unique pharmaceutical agent in its class. This specific design contributes to targeted and effective modulation of XO activity, highlighting its significance in the treatment of conditions associated with abnormal purine metabolism, such as gout. Febuxostat demonstrates remarkable specificity in its effect on enzymes related to purine or pyrimidine metabolism [66]. Although it effectively inhibits XO, its influence on other crucial enzymes in this metabolic pathway, such as purine nucleoside phosphorylase, adenosine deaminase and pyrimidine nucleoside phosphorylase, is minimal. This targeted action is significant, as it allows for the selective modulation of XO without interfering with the activities of other enzymes associated with purine or pyrimidine metabolism. Notably, the pharmacological profile of febuxostat underscores its ability to address specific targets, providing a nuanced and tailored approach for the treatment of conditions related to aberrant purine metabolism, such as gout.

Upon oral administration, febuxostat exhibits high absorption, with approximately 85% of the administered dose readily absorbed [67]. Interestingly, doses exceeding 120 mg/day resulted in a greater than dose-proportional increase in the area under the curve (AUC). This phenomenon is attributed to a reduction in renal clearance of the conjugated form, coupled with an elevation in biliary excretion and enterohepatic recirculation. The presence of food, particularly high-fat meals, impedes the absorption of febuxostat, leading to a decrease in the AUC by 16–19%. At steady state, Febuxostat demonstrated a volume of distribution of 0.7 L/kg. It is worth noting that the drug exhibits high binding affinity, with 99.2% binding to circulating plasma proteins such as albumin [68]. Febuxostat undergoes hepatic metabolism within the cytochrome P450 enzyme system, which results in the formation of acylglucuronide metabolites. This metabolic process predominantly involves conjugation facilitated by uridine diphosphate-glucuronosyltransferase. The elimination of febuxostat occurs predominantly through the renal pathways, with a mean half-life (*t*_1/2_) of 9.1 h observed for a 120-mg dose [69]. This comprehensive understanding of the pharmacokinetic profile of febuxostat provides valuable insights into its absorption, distribution, metabolism and elimination dynamics, aiding its effective clinical application. *In vitro* studies showed a minimal impact of febuxostat on various isoenzymes, with a weak inhibitory effect on CYP2D6. No significant interactions were observed between colchicine and indomethacin. Coadministration with antacids slightly reduced the *C*_max_ of febuxostat by 32%, but the AUC was unaffected [69]. Although no formal studies have been conducted, caution is advised when using febuxostat with theophylline, mercaptopurine or azathioprine because of the potential risks of increased drug levels and toxicity. Febuxostat offers flexibility of administration without the need to consider food intake or the concurrent use of antacids. Patients with mild or moderate renal impairment, as well as those with mild to moderate hepatic impairment, do not require dosage adjustments when using febuxostat. However, it is important to note that febuxostat is contraindicated in patients undergoing treatment with azathioprine, mercaptopurine or theophylline. This caution arises from the potential of concurrent administration of febuxostat to elevate the plasma levels of these specific drugs [70]. To prevent any adverse interactions, the concomitant use of febuxostat with azathioprine, mercaptopurine or theophylline should be avoided. This precautionary measure ensures the optimal and safe use of febuxostat in clinical settings.

O’Dell et al. conducted a trial indicating that allopurinol is as effective as febuxostat in reducing flares in individuals with gout [71]. This study emphasizes that both urate-lowering therapies, when applied in a titrate-to-target approach, are highly effective in achieving uniform serum urate goals. This efficacy extends to participants with stage 3 chronic kidney disease, a prevalent comorbidity in gout. Importantly, the study found no indications that febuxostat elevates cardiovascular morbidity or overall mortality when compared to allopurinol. The investigation conducted by Zhang et al. did not achieve the primary objective of establishing the non-inferiority of febuxostat at a dose of 40 mg/day in comparison to allopurinol at a dosage of 300 mg/day, as determined by the proportion of participants with serum UA levels equal to or below 6.0 mg/dL at the 24-week mark [72]. Nevertheless, febuxostat at 60 and 80 mg/day showed superiority when compared to allopurinol at 300 mg per day, as evidenced by the percentage of participants with serum UA levels equal to or below 6.0 mg/dL at the 16-week and 24-week intervals, respectively. Peng et al. indicated that febuxostat demonstrated superiority over allopurinol in maintaining a sustained reduction in serum UA levels among patients with chronic kidney disease [73]. However, there were no discernible differences in renal function changes between patients who received either febuxostat or allopurinol in a typical clinical setting. It is crucial to closely monitor serum creatinine and UA levels in patients undergoing XO inhibitor therapy to promptly identify acute kidney injury and prevent the deterioration of renal function. To establish cost-effective practices, they suggested that further long-term follow-up studies are essential to assess variances in renal outcomes between febuxostat and allopurinol in patients with chronic kidney disease and hyperuricaemia. The study conducted by Pawar et al. provided reassurance for patients intolerant to allopurinol requiring febuxostat treatment, as it did not detect elevated cardiovascular or all-cause mortality, even in individuals with a history of cardiovascular disease [74]. The strengths of the study include its incident new-user and active-comparator design, employing propensity score matching to minimize confounding factors. These findings are applicable to a broad population of elderly patients with gout. The primary as-treated approach addresses non-adherence bias, which is a limitation of the CARES trial. However, residual confounding remains a possibility, and misclassification bias may arise from reliance on diagnostic codes for participant eligibility, covariates and outcome identification. Several other studies on febuxostat are presented in Table 1.

**Table 1.** Use of febuxostat against several other complications with a specific mechanism of action.

Specific functions	Chemical agent used	Mode of action	References
Febuxostat suppresses endoplasmic (ER) stress	Tunicamycin	Through upregulation of SIRT1-AMPK-HO-1/TRX expression	[75]
Reduces NLRP3-dependant inflammations	Nigericin, MSU	MitoROS independent activation of purine salvage pathways and restoration of cellular ATP/bioenergetics	[76]
Protective effect against ulcerative colitis	Acetic acid	Inhibiting inflammatory mediators (NF-κB) and oxidative stress moderators	[77]
Prevent necrosis of the skin flap	–	Reduced expression of interleukin-1β inhibits oxidative stress and inflammation due to ischemia-reperfusion.	[78]
Prevent ROS-dependant osteoclastic bone loss	RNKL, Dox	ROS scavenging	[79]
Therapeutic potential against allergic rhinitis	Histamine	Inhibit inflammation regulating transcriptional factor KLF6	[79]
Suppress adipogenesis	Hydrogen peroxide	Controlling ROS production and Nrf2 activation	[80]
Relieves renal injury	Arsenic trioxide	Inhibiting the expression of TLR4, caspase-1, ASC and NLRP3	[81]
Protects brain post intracerebral haemorrhage	–	Regulating LncRNA	[82]
Therapeutic efficacy for Parkinson’s disease	1-Methyl-4-phenyl-pyridine	Suppress the JNK/NF-κB signalling pathways	[83]

Abbreviations: SIRT1, silent mating type information regulation 2 homology 1; AMPK, AMP activated protein kinase; HO-1, [heme oxygenase-1; TRX, thioredoxin; NLRP3, nucleotide-binding oligomerization domain (NOD), Leucine-rich repeat (LRR), Pyrin domain-containing protein 3; MSU, monosodium urate; MitoROS, mitochondrial ROS; NF-κB, nuclear factor kappa-light-chain-enhancer of activated B cells; RNKL, receptor activator of NF-κB ligand; Dox, doxorubicin; KLF6, Kruppel-like-factor-6; Nrf2, nuclear factor erythroid-2-related factor 2; TLR4, toll-like receptor 4; ASC, apoptosis-associated speck-like protein containing a CARD (caspase recruitment domain); LncRNA, long non-coding RNA; JNK, c-Jun NH_2_-terminal kinase.

## 5. UA production on hypertension

**Figure 3.**
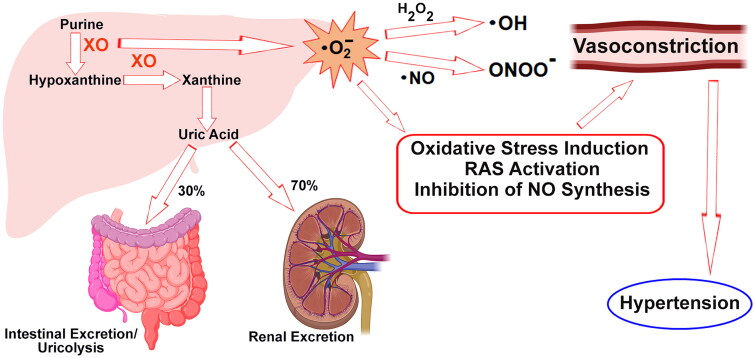
Important metabolic pathways that relate hypertension and serum UA level.

The correlation between serum UA levels and elevated blood pressure or hypertension in humans has been established. An illustrative case comes from a cross-sectional study, where it was found that each incremental increase of 1 mg/dL in serum UA was associated with an approximately 20% greater prevalence of hypertension within a general population that was not undergoing treatment for hyperuricaemia and hypertension [84,85]. Furthermore, when examining data from longitudinal cohort studies, it becomes apparent that individuals with asymptomatic hyperuricaemia, devoid of any accompanying health issues, are more likely to develop hypertension over time. This finding suggests a significant link between elevated UA levels and the subsequent onset of high blood pressure. Moreover, the influence of hyperuricaemia extends beyond merely predicting hypertension. It also contributes to the transition from prehypertension, a precursor stage, to fully manifest hypertension [86]. This highlights the progressive role of UA in the continuum of blood pressure elevation. In light of these comprehensive observations, the reduction of serum UA levels has emerged as a particularly intriguing and promising therapeutic approach in the realm of hypertension management. By addressing the role of UA in predicting and contributing to hypertension, interventions aimed at lowering UA levels may have substantial potential in mitigating the risk and progression of high blood pressure. Research has shown that increased serum UA levels can activate the renin-angiotensin system (RAS) [87]. This activation occurs either through the direct inhibition of nitric oxide (NO) synthesis in the juxtaglomerular apparatus or indirectly by stimulating the proliferation of smooth muscle cells in the wall of the afferent arteriola (Figure 3). These processes ultimately lead to a reduction in renal perfusion, highlighting the intricate mechanisms through which elevated serum UA levels may affect the regulatory systems involved in blood pressure and renal function.

Hyperuricaemia contributes to arterial stiffness via two primary mechanisms. In the first scenario, elevated levels of serum UA directly induce arteriosclerosis. This occurs as macrophages engulf urate crystals, triggering activation of the NOD-like receptor NLRP3 inflammasome [88]. Consequently, the NLRP3 inflammasome pathway plays a crucial role in interleukin-1β secretion from MSU-stimulated human macrophages. This secretion, which occurs in a post-translational modification-related manner, leads to inflammation and heightened collagen production, ultimately fostering the progression of arteriosclerosis. Atherosclerosis is strongly correlated with arterial stiffness at various locations in the vascular network. In the second mechanism, UA induces oxidative stress within the cells and mitochondria, diminishing the bioavailability of endothelial NO. This process also stimulates the intracellular RAS [89]. The combined effects of intracellular and mitochondrial oxidative stress reduced NO availability and activated RAS, collectively contributing to the development of arterial stiffness (Figure 3). Hyperuricaemia is widely recognized as a biomarker for XO activation, a process that releases oxidants during UA generation. The increased production of oxidants, particularly superoxide, contributes to endothelial dysfunction. Human blood vessels express the urate transporter GLUT9, which is a key player in the absorption of UA into the bloodstream. This absorption triggers inflammation, dephosphorylation of endothelial nitric oxide synthase (eNOS) in the vasculature and oxidative stress [90]. NO plays a crucial role in regulating vascular tone and arterial stiffness. Any reduction in NO bioavailability or disproportion in its reduced production can lead to endothelial dysfunction, subsequently resulting in elevated blood pressure. UA within the blood vessels sets in motion a cascade of events, including inflammation, which ultimately leads to arteriosclerosis and arterial stiffness. In this intricate pathway, the interplay between UA, oxidative stress and NO availability is a critical determinant of vascular health and the development of cardiovascular complications [91].

Based on both *in vitro* and *in vivo* data collected by Lee et al. dysregulation of the asymmetric dimethylarginine (ADMA)/DDAH-2 (dimethyl arginine dimethylaminotransferases-2) axis plays a crucial role in the activation of Nicotinamide Adenine Dinucleotide Phosphate (NADPH) oxidase/ROS signalling, endothelial cell dysfunction, inflammation and the hastening of atherosclerosis induced by hyperuricaemia [92]. These findings reveal a novel molecular mechanism that underscores the adverse effects of hyperuricaemia on atherosclerosis development. Furthermore, they proposed a connection between UA metabolism and the ADMA/DDAH-2-component related to the cardiovascular system. Wang et al. found a link between a higher serum UA level and serum creatinine ratio and an increased risk of hypertension [93]. Their analyses suggested that blood lipid concentration, body mass index, blood pressure, high-sensitivity C-reactive protein and blood glucose concentration may act as possible mediators of this association. This highlights the possible role of these factors in the observed association between serum UA and serum creatinine levels and cardiovascular disease risk. The research carried out by Tian et al. revealed that an elevated risk of myocardial infarction is specifically linked to consistently high levels of serum UA [94]. Additionally, the study confirmed that hypertension plays a mediating role in the association between variations in serum UA levels and the occurrence of myocardial infarction.

It is crucial for health care professionals and public health practitioners to recognize that monitoring the longitudinal trends of both serum UA and hypertension could contribute to reducing the risk of myocardial infarction. Cardio-nephro-metabolic disorders must be evaluated in a diverse sample that is representative of the general population in order to clarify the relationship between UA levels and these disorders. Clarification of serum UA threshold values is crucial for identifying elevated UA levels associated with heightened risk. In order to address this need, the Uric Acid Right for Heart Health (URRAH) project has been specifically designed [95–97]. An essential aim of this study is to determine the level of uricaemia that, in a general Italian population, is associated with a significant increase in the independent risk of cardiovascular disease. There is a direct relationship between serum UA and serum creatinine levels (SUA/sCr), and this ratio provides a more comprehensive assessment than serum UA alone when it comes to predicting cardiovascular risk since it considers the influence of renal function, which is closely related to serum UA levels. In the URRAH population, a threshold indicating cardiovascular risk has recently been established. An SUA/sCr ratio greater than 5.35 units has been shown to be an independent predictor of mortality from cardiovascular disease among men and women [98]. Table 2 summarizes a few additional studies on the mechanism underlying the relationship between hypertension and uricaemia.

**Table 2.** Proposed mechanism related to the connection between UA level elevation and hypertension.

Model/method used	Mechanism involved	References
Genotyped-based studies on 2769 European populations	Hypertension is associated with the SNPs in XAN oxidoreductase genes	[99]
312 participants from Bangladeshi adults	XO affects the pathophysiology of hypertension, generating ROS	[100]
Unilaterally nephrectomized Sprague-Dawley male rats	Endothelin-1 activated XO functions and mitochondrial oxidative phosphorylation to promote vascular ROS generation and hypertension	[101]
Patients with hypertension and renal dysfunction	XO-based ROS generation to the progression of hypertension	[102]
Neonatal rats	XO-derived superoxide	[103]
Population-based cohort studies on nondiabetic 271 subjects	XOR activity and ROS generation are the key factors for hypertension in nondiabetics	[104]
192 volunteers not taking medication to treat hyperuricaemia	ROS-based oxidative stress due to UA without XOR involvement	[105]
Sprague-Dawley rats	Hyperuricaemia induces oxidative stress, and the RAS is involved	[106]
1548 participants with preeclampsia	UA and superoxide dismutase are responsible for hypertension	[107]
123 participants	Higher levels of UA lower nitrite concentrations	[108]
17 pregnant women having preeclampsia and 14 normal pregnant women	Higher serum UA concentrations elevate serum hydrogen peroxide levels and oxidative stress	[109]

## 6. Elevated UA on atrial fibrillation

The prevalence and incidence of atrial fibrillation (Afib) are increasing worldwide, making it one of the most common cardiac arrhythmias in the elderly. Numerous investigations have identified a link between heightened serum UA levels and a higher likelihood of AFib [91]. However, it remains uncertain whether UA actively contributes to the mechanisms underlying the onset and persistence of AFib. The association between UA and AFib can be attributed to features such as oxidative stress, related inflammation, activation of the renin–angiotensin–aldosterone system and endothelial dysfunction [110]. The association between elevated UA levels and the progression or development of AFib may be explained by the fact that each of these elements has been demonstrated to contribute to atrial remodelling. In a cross-sectional study of a large Japanese general population conducted by Kawasoe et al. a significant association was found between serum UA levels and AFib in both sexes [111]. Ding et al. found that elevated levels of UA were associated with an increased risk of serious health problems after AFib diagnosis in middle adulthood [112]. It may also be possible, as they concluded, to link high levels of UA directly to the development of AFib via mechanisms other than CVD or cardiovascular risk factors, which may increase the risk of AFib. Based on clinical and experimental evidence, hyperuricaemia has been associated with an elevated incidence of AFib, as shown by Deng et al. [113]. They also demonstrated in a number of small *in vitro* and *in vivo* studies that hyperuricaemia, both at low and high UA concentrations, may play a crucial role in the pathogenesis of AFib by activating oxidative stress, stimulating inflammation, triggering fibrosis and immune responses.

## 7. Disease-associated with UA in the kidneys

Whether lowering a patient’s serum UA level in the case of gout will have an effect on kidney damage or cardiovascular events has been controversial. It is believed that urate crystals accumulate not only in the joints but also in several tissues [114]. The collecting ducts of the kidneys are one of the most common locations of urate crystals. The concentration of UA in urine increases its acidity, facilitating the crystallization of urate as the urine becomes more acidic. The adhesion of some crystals of urate to the tubular epithelium can lead to local inflammation and rupture of the tubular wall, which allows the crystals to escape into the interstitial space. The process of inflammation may trigger localized macrophage infiltration as a result of this process. Individuals with gout often excrete substantially less UA than normal, especially after consuming meals high in purines and fructose. In addition to preventing recurrent gout attacks, emerging evidence suggests that urate crystals play a direct role in atherosclerosis. Urate crystals are found in the bloodstream of a substantial percentage of individuals with gout, ranging from 75% to 86% [115]. Additionally, almost 30% of subjects diagnosed with gout exhibit the presence of these crystals, specifically in their coronary arteries, with atherosclerotic plaques appearing to be the primary location. These lesions seem to be at risk of expanding or rupturing because urate crystals activate inflammasomes, similar to cholesterol crystals. The link between hyperuricaemia, gout and cardiovascular mortality may be explained by these findings. In addition, crystal deposits of urate can trigger both local and systemic inflammation, which contribute to kidney disease and cardiovascular events, in addition to local inflammation.

## 8. XO inhibitors on hypertension

Febuxostat and allopurinol, inhibitors of XO, showed comparable efficacy in averting gout attacks in individuals with gout. However, febuxostat has a strong potential to lower blood UA levels [116]. Allopurinol competes with XAN to bind to XO as a XAN analog, and the active metabolite of this compound competes with HPX, consequently impeding the production of UA. In contrast, febuxostat selectively blocked XO activity without further involvement of metabolites. Moreover, UA triggers the growth of vascular smooth muscle cells and plays a significant role as an inflammatory agent. The activity of XO produces ROS, such as superoxide, H_2_O_2_ and hydroxyl radicals [117]. These ROS contribute to tissue damage and impairment of NO function, ultimately leading to endothelial dysfunction, an initial stage of atherosclerosis, and vascular damage. Allopurinol inhibits XO activity, thereby decreasing ROS levels and increasing the availability of NO for vascular smooth muscle relaxation.

**Figure 4.**
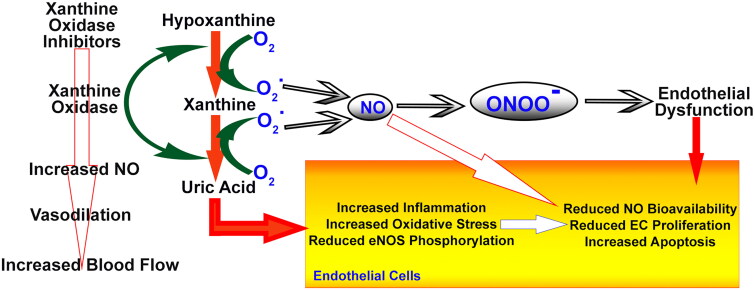
The way XO inhibitors affect hypertension.

NO synthesis by the vascular endothelium is vital for the maintenance of vasodilator tone. Aside from its role in vasodilation, endothelium-derived NO has profound physiological effects that influence cardiovascular homeostasis in profound ways. The ability of endothelium-derived NO to inhibit smooth muscle cell proliferation is crucial for preventing arterial wall thickening, which is a process associated with atherosclerosis. Additionally, NO plays a key role in preventing leukocyte adhesion to the vascular surface, contributing to an overall anti-inflammatory environment within the blood vessels by reducing platelet aggregability, thereby diminishing the risk of blood clot formation [118]. Various aspects of cardiovascular health are regulated by endothelium-derived NO through multifaceted actions. The impaired bioavailability of NO originating from the endothelium may contribute to atherosclerosis. Decreased NO activity has been identified as an indicator of endothelial dysfunction in individuals with established atherosclerosis risk factors, such as hypercholesterolemia and arterial hypertension [119]. This highlights the intricate relationship between NO synthesis, endothelial function and cardiovascular diseases, emphasizing the importance of maintaining healthy endothelium. Endothelial dysfunction and increased degradation of NO are linked in numerous animal models of hypercholesterolemia and hypertension, according to numerous studies. As scavengers of endothelium-derived NO, superoxide anions are integral to this degradation process. It is generated by a variety of pathways involving enzymes and nonenzymes. In addition to the activation of circulating neutrophils, the XO system plays a major role in the production of superoxide anions within the vascular endothelium. Endothelial cells were exposed to superoxide anions using the XO system. XO can be inhibited with inhibitors such as febuxostat, allopurinol and oxypurinol, which are structurally similar to XAN [120]. These XO inhibitors bind to XO and prevent radical formation and UA production. This suggests that superoxide anions, primarily derived from the XO system, may be responsible for the reduced bioavailability of NO in patients with hypercholesterolemia and essential hypertension (Figure 4). Hypercholesterolemia and hypertension are cardiovascular conditions associated with endothelial dysfunction, which can be addressed through therapeutic interventions targeting superoxide anions and NO.

## 9. XO inhibitor-based nanomedicine

**Figure 5.**
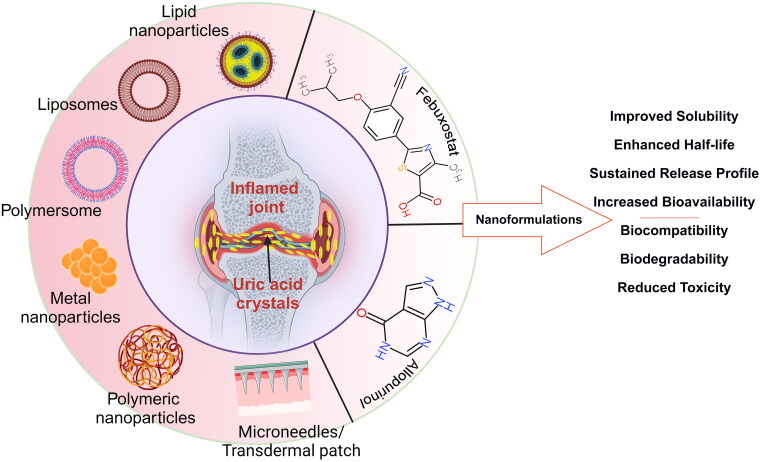
Nanostructures involved in the formulations of allopurinol and febuxostat.

In an effort to bridge the gap between biological and physical sciences, nanotechnology has proven to be extremely successful in utilizing nanostructures and nanophases across a wide range of scientific fields [121–123]. This is particularly evident in areas such as nanomedicine and nano-based drug delivery systems, where the focus is on the significant potential that can be derived from such nanoparticles. Significant advancements have been made in nanomedicine through the use of therapeutic agents on the nanoscale, including drug delivery, biosensing and a wide variety of applications in tissue engineering [124,125]. Nanoparticles are composed of molecules or atoms engineered at the atomic level. Nanoscale particles exhibit distinct structural, chemical, mechanical, magnetic, electrical and biological properties [25,126]. This allows them to manoeuvre within the human body more freely than bulk materials do. By addressing issues ranging from biodistribution to intracellular trafficking, nanotechnology can address some of the drawbacks of traditional delivery methods. Specific cell targeting, molecular transport to diseased cells or specific organelles, and other innovative approaches can be used to accomplish this goal. As a result of nanoparticles, enclosed payloads are more stable and solubilized, membranes are more easily crossed, and circulation times are extended, thus enhancing safety and efficacy (Figure 5) [24,127].

Fuxostat is classified as class 2 by the biopharmaceutical classification system because of its low solubility in water and weak acidity in nature, with a pKa of approximately 3.08. Therefore, Bhatt et al. encapsulated febuxostat in a nano-based lipid carrier composed of oleic acid and stearic acid [128]. The optimized formulations were engineered using a combination of the homogenization process and bath sonication techniques and showed an encapsulation efficiency of more than 80 ± 2.3%. The size of the formulated nanostructures was restricted to less than 229 nm, demonstrating the release of febuxostat with zero-ordered kinetics, making it ideal for oral delivery for the treatment of chronic gout. Gurumukhi et al. reported febuxostat-loaded nanoemulsions to improve bioavailability and membrane permeability [129]. Febuxostat loaded in the microemulsion showed increased permeability in the artificial membrane model and everted-gut sac system. An approximately 2.5-fold increase in bioavailability was observed in Wistar rat models, and the formulation was stable for more than 90 days at room temperature (25 °C) and 4 °C. To overcome the poor gastrointestinal absorption and bioavailability of febuxostat, Al-Amodi et al. incorporated this XO inhibitor into a self-emulsifying nano-drug delivery system composed of several essential oils, surfactants and lipids [130]. The group established the efficacy of these formulations in the management of gout using several *in vitro* and *in vivo* studies.

The limited solubility in aqueous media and susceptibility to enzymatic breakdown in the liver or intestine limit the oral delivery of febuxostat. Simultaneously, the plasma concentration of febuxostat was affected by food intake. Therefore, Singh et al. proposed transdermal delivery of febuxostat using niosomal gel formulations [131]. The formulations were prepared using thin-film hydration techniques, and Span-60 and Tween-20 were used as essential components. They demonstrated an extended drug permeation of approximately 80.4% in *in vitro* and *in vivo* studies using rabbit models. A transdermal drug delivery system for febuxostat was developed by Patel et al. to improve patient compliance and bioavailability [132]. Using a bottom-up approach and micromoulding, febuxostat cubosomes were incorporated into a microneedle to improve skin penetration. In this process, lipids and polyvinyl alcohol are used along with specific organic and aqueous phases. They concluded that the nano-patch loaded with febuxostat in cubosomes maintained UA levels better than other reported formulations in rats. As a member of the Biopharmaceutics Classification System class-2 drugs, allopurinol encounters solubility issues, impacting its bioavailability and reducing its plasma half-life. Ali et al. used lipid-based nanocarriers in their study to increase bioavailability and improve efficacy and safety for the *in vivo* use of formulated allopurinol [133]. Drug-loaded emulsions were prepared using oleic acid, stearic acid and Tween-20 surfactants. The formulations showed a sustained-release profile with reduced toxicity and better anti-gout activity than the conventional allopurinol treatment. A few more nanoformulated applications of allopurinol and febuxostat are given in Table 3.

**Table 3.** Applications of nanoformulated XO inhibitors.

Drugs used	Specific nanoformulations	Treatment/purpose	References
Allopurinol	Low MW chitosan nanoparticles	Hyperuricemic-connected nephrolithiasis management	[134]
Allopurinol	BSA nanoparticles	Hyperuricemic-linked nephrolithiasis maintenance	[135]
Allopurinol	Thioctic acid-loaded lipid nanocarriers	Gout and nephrotoxicity treatment	[136]
Febuxostat	Hydroxypropyl methylcellulose and α-D-Tocopherol-PEG1000-succinate stabilized nanosuspension	Increased oral bioavailability	[137]
Febuxostat	B-cyclodextrin based nanosponge	Improvement of oral bioavailability	[138]
Febuxostat	Chitosan nanoparticles	Increased encapsulation efficiency and bioavailability to alter the pharmacokinetics with sustained release	[139]
Febuxostat	Chitosan-coated tin dioxide-based nanosphere	Increase intestinal absorption and bioavailability	[140]
Allopurinol	Glutaraldehyde-based polymerized albumin microparticles	Management of ischemic stroke and hypertension	[141]
Allopurinol	Chitosan alginate nanohydrogels	Modulate oxidative stress and decrease inflammation	[142]
Febuxostat	Nanoengineered lipid carrier	Modified release profile, improved dissolution, bioavailability minimizing the side effect	[143]
Febuxostat	Ethosomes made of ethanol and soya lecithin	Better transdermal delivery system for gout management	[144]

Abbreviations: BSA, bovine serum albumin; PEG, polyethylene glycol.

## 10. Coadministration of hyperuricemic and hypertensive drugs

An increased concentration of UA in blood serum is closely associated with the inception and progression of hypertension. Therefore, treatment to lower serum UA levels causes a reduction in blood pressure or hypertension. XO inhibitors constitute the primary category of hyperuricemic medications and have the potential to reduce hypertension. Elevated UA levels mainly affect hypertension in two phases [145]. Initially, UA can prompt acute vasoconstriction through the activation of the renin–angiotensin–aldosterone system. Second, the absorption of serum UA into vascular smooth muscle cells results in cellular proliferation and subsequent arteriolosclerosis, compromising the compliance of resistant vessels and impeding blood pressure-induced natriuresis. Hence, hypertension is a common comorbidity in individuals with gout. This considerable comorbidity burden may arise from either the co-pathogenesis of the two conditions or renal alterations in hypertension, resulting in diminished urate excretion [146]. Research indicates that the occurrence of hypertension is autonomously linked to the likelihood of developing gout, which is primarily attributable to lowered renal blood circulation, heightened renal and systemic vascular resistance and diminished renal excretion of urate.

Certain antihypertensive medications have been shown to elevate serum UA levels, thereby potentially contributing to an elevated risk of gout. Beyond the well-established associations between diuretic-induced hyperuricaemia and related gout, it has been perceived that the use of β-blockers can lead to an increase in serum UA levels. This phenomenon has been documented in short-term trials, suggesting a link between β-blocker use and the modulation of UA concentrations, adding another dimension to the understanding of how certain antihypertensive drugs may influence the risk profile for gout [147]. The co-crystallization of drugs represents an innovative mechanism for facilitating effective pharmaceutical combinations in therapy. Ganesan et al. synthesized a drug-drug co-crystal, focusing on combining a hypertension therapeutic, telmisartan and a hyperuricaemia drug, febuxostat, in a 1:1 molar ratio [148]. This co-crystal was prepared using a solvent evaporation method, as outlined in their research work. This approach represents a noteworthy endeavour in exploring novel formulations through co-crystallization, potentially offering enhanced therapeutic outcomes through the combination of these two pharmacologically relevant drugs. A febuxostat-telmisartan co-crystal (120 mg) was composed of approximately 46 mg of febuxostat and 74 mg of telmisartan [148]. These quantities align with the intended range, mirroring the standard doses of febuxostat used in clinical practice, which is typically 40–80 mg/day, and telmisartan, which is usually prescribed at doses ranging from to 40–80 mg/day. This formulation was designed strategically to optimize the combined pharmacological effects within the established therapeutic ranges for both constituent drugs. This groundbreaking concept holds the potential to provide a significant breakthrough for individuals with both hyperuricaemia and hypertension.

## 11. Conclusion and future perspective

Considering the involvement of XO in diverse pathological conditions, it becomes apparent that targeted inhibition of XO could lead to a wide-ranging therapeutic approach for ailments such as gout or hyperuricaemia, inflammation and oxidative damage. Therefore, XO inhibitors have emerged as promising agents with substantial potential for the treatment of various medical conditions. Hyperuricaemia and gout primarily manifest as excessive UA production. XO inhibitors have demonstrated exceptional efficacy in reducing UA levels in the bloodstream and specific peripheral tissues. Their role in the management of hypertension-related disorders is also discussed in this review, which can help physicians during prescription. Studies have clearly demonstrated that nanoformulations of allopurinol and febuxostat resolved several challenges regarding the poor solubility and bioavailability of these potent drugs during the management of diseased conditions.

The primary objective of this review was to caution clinicians regarding prescribing medications for hypertension in patients with hyperuricaemia. Instead of approaching these conditions separately, there is a potential benefit of simultaneously addressing hyperuricaemia by using XO inhibitors. These inhibitors can manage both conditions concurrently, providing a more holistic approach to treatment. In the realm of nanotechnology, there is clear potential for reducing the dosage of XO inhibitors through enhancement of bioavailability and circulation half-life. As time advances, more refined formulations are expected to be created, and researchers will continue to develop improved nanoformulations. During the engineering of new nanoformulations, the choice of materials and their biocompatibility are the main focus, along with their cost-effective formulations. Polymer chemistry is growing rapidly, and several biocompatible and biodegradable polymers have been reported to be ideal carriers for transporting medically active components. XO inhibitors can be encapsulated to enhance their circulation half-life and other therapeutic benefits to control serum UA and blood pressure. Another approach to the co-crystal concept was presented in the previous section. More co-crystal synthesis in a balanced way will be the game changer and will help medical practitioners easily generate prescriptions for those who are suffering from both hyperuricaemia and hypertension. These advancements aim to overcome the current challenges faced by allopurinol and febuxostat, promising a more effective and efficient approach to their application.

## Data Availability

All the data are available in the manuscript.

## References

[1] Gonsalez SR, Cortês AL, Silva R, et al. Acute kidney injury overview: from basic findings to new prevention and therapy strategies. Pharmacol Ther. 2019;200:1–20. doi: 10.1016/j.pharmthera.2019.04.001. 30959059 PMC10134404

[2] Bagga HS, Chi T, Miller J, et al. New insights into the pathogenesis of renal calculi. Urol Clin North Am. 2013;40(1):1–12. doi: 10.1016/j.ucl.2012.09.006. 23177630 PMC4165395

[3] Devarajan A. Cross-talk between renal lithogenesis and atherosclerosis: an unveiled link between kidney stone formation and cardiovascular diseases. Clin Sci. 2018;132(6):615–626. doi: 10.1042/CS20171574. 29559506

[4] Gambaro G, Croppi E, Coe F, et al. Metabolic diagnosis and medical prevention of calcium nephrolithiasis and its systemic manifestations: a consensus statement. J Nephrol. 2016;29(6):715–734. doi: 10.1007/s40620-016-0329-y. 27456839 PMC5080344

[5] Low RK, Stoller ML. Uric acid–related nephrolithiasis. Urol Clin North Am. 1997;24(1):135–148. doi: 10.1016/s0094-0143(05)70359-1. 9048857

[6] Shekarriz B, Stoller ML. Uric acid nephrolithiasis: current concepts and controversies. J Urol. 2002;168(4 Pt 1):1307–1314. doi: 10.1016/S0022-5347(05)64439-4. 12352383

[7] Abate N, Chandalia M, Cabo-Chan AV, et al. The metabolic syndrome and uric acid nephrolithiasis: novel features of renal manifestation of insulin resistance. Kidney Int. 2004;65(2):386–392. doi: 10.1111/j.1523-1755.2004.00386.x. 14717908

[8] de Oliveira EP, Burini RC. High plasma uric acid concentration: causes and consequences. Diabetol Metab Syndr. 2012;4(1):12. doi: 10.1186/1758-5996-4-12. 22475652 PMC3359272

[9] Maiuolo J, Oppedisano F, Gratteri S, et al. Regulation of uric acid metabolism and excretion. Int J Cardiol. 2016;213:8–14. doi: 10.1016/j.ijcard.2015.08.109. 26316329

[10] Borghi C, Agabiti-Rosei E, Johnson RJ, et al. Hyperuricaemia and gout in cardiovascular, metabolic and kidney disease. Eur J Intern Med. 2020;80:1–11. doi: 10.1016/j.ejim.2020.07.006. 32739239

[11] Johnson RJ, Bakris GL, Borghi C, et al. Hyperuricemia, acute and chronic kidney disease, hypertension, and cardiovascular disease: report of a scientific workshop organized by the national kidney foundation. Am J Kidney Dis. 2018;71(6):851–865. doi: 10.1053/j.ajkd.2017.12.009. 29496260 PMC7286363

[12] Bruce SP. Febuxostat: a selective xanthine oxidase inhibitor for the treatment of hyperuricemia and gout. Ann Pharmacother. 2006;40(12):2187–2194. doi: 10.1345/aph.1H121. 17132810

[13] Suzuki S, Yoshihisa A, Yokokawa T, et al. Comparison between febuxostat and allopurinol uric acid-lowering therapy in patients with chronic heart failure and hyperuricemia: a multicenter randomized controlled trial. J Int Med Res. 2021;49(12):3000605211062770. 03000605211062770. doi: 10.1177/03000605211062770. 34914568 PMC8689623

[14] Hu M, Tomlinson B. Febuxostat in the management of hyperuricemia and chronic gout: a review. Ther Clin Risk Manag. 2008;4(6):1209–1220. 19337428 10.2147/tcrm.s3310 PMC2643102

[15] Beara-Lasic L, Pillinger MH, Goldfarb DS. Advances in the management of gout: critical appraisal of febuxostat in the control of hyperuricemia. Int J Nephrol Renovasc Dis. 2010;3:1–10. doi: 10.2147/ijnrd.s5563. 21694922 PMC3108781

[16] Borgi L, McMullan C, Wohlhueter A, et al. Effect of uric acid–lowering agents on endothelial function. Hypertension. 2017;69(2):243–248. doi: 10.1161/HYPERTENSIONAHA.116.08488. 28028194 PMC5233648

[17] Su H-y, Yang C, Liang D, et al. Research advances in the mechanisms of hyperuricemia-induced renal injury. Biomed Res Int. 2020;2020:5817348. doi: 10.1155/2020/5817348. 32685502 PMC7336201

[18] Cicero AFG, Fogacci F, Cincione RI, et al. Clinical effects of xanthine oxidase inhibitors in hyperuricemic patients. Med Princ Pract. 2021;30(2):122–130. doi: 10.1159/000512178. 33040063 PMC8114083

[19] Girigoswami K, Pallavi P, Girigoswami A. Intricate subcellular journey of nanoparticles to the enigmatic domains of endoplasmic reticulum. Drug Deliv. 2023;30(1):2284684. doi: 10.1080/10717544.2023.2284684. 37990530 PMC10987057

[20] Mary Lazer L, Sadhasivam B, Palaniyandi K, et al. Chitosan-based nano-formulation enhances the anticancer efficacy of hesperetin. Int J Biol Macromol. 2018;107(Pt B):1988–1998. doi: 10.1016/j.ijbiomac.2017.10.064. 29032208

[21] Vedhanayagam M, Nidhin M, Duraipandy N, et al. Role of nanoparticle size in self-assemble processes of collagen for tissue engineering application. Int J Biol Macromol. 2017;99:655–664. doi: 10.1016/j.ijbiomac.2017.02.102. 28274865

[22] Draviana HT, Fitriannisa I, Khafid M, et al. Size and charge effects of metal nanoclusters on antibacterial mechanisms. J Nanobiotechnol. 2023;21(1):428. doi: 10.1186/s12951-023-02208-3. PMC10648733 37968705

[23] Pourmadadi M, Mahdi Eshaghi M, Ostovar S, et al. Innovative nanomaterials for cancer diagnosis, imaging, and therapy: drug delivery applications. J Drug Deliv Sci Technol. 2023;82:104357. doi: 10.1016/j.jddst.2023.104357.

[24] Thirumalai A, Girigoswami K, Pallavi P, et al. Cancer therapy with iRGD as a tumor-penetrating peptide. Bull Cancer. 2023;110(12):1288–1300. doi: 10.1016/j.bulcan.2023.08.009. 37813754

[25] Thirumalai A, Harini K, Pallavi P, et al. Bile salt-mediated surface-engineered bilosome-nanocarriers for delivering therapeutics. Nanomed J. 2024;11(1):1–12.

[26] Dragicevic N, Predic-Atkinson J, Nikolic B, et al. Nanocarriers in topical photodynamic therapy. Expert Opin Drug Deliv. 2024;21(2):279–307. doi: 10.1080/17425247.2024.2318460. 38349540

[27] Pemula G, Anand AV, Karthick H, et al. Nanostructured proteins for delivering drugs to diseased tissues. Bioinspired Biomim Nanobiomaterials. 2023;12(3):115–129.

[28] Shurfa MK, Girigoswami A, Sakthi Devi R, et al. Combinatorial effect of doxorubicin entrapped in alginate-chitosan hybrid polymer and cerium oxide nanocomposites on skin cancer management in mice. J Pharm Sci. 2023;112(11):2891–2900. doi: 10.1016/j.xphs.2023.08.014. 37611665

[29] Naahidi S, Jafari M, Edalat F, et al. Biocompatibility of engineered nanoparticles for drug delivery. J Control Release. 2013;166(2):182–194. doi: 10.1016/j.jconrel.2012.12.013. 23262199

[30] Otto DP, Otto A, de Villiers MM. Differences in physicochemical properties to consider in the design, evaluation and choice between microparticles and nanoparticles for drug delivery. Expert Opin Drug Deliv. 2015;12(5):763–777. doi: 10.1517/17425247.2015.988135. 25516397

[31] Day Richard O, Kamel B, Kannangara Diluk RW, et al. Xanthine oxidoreductase and its inhibitors: relevance for gout. Clin Sci. 2016;130(23):2167–2180. doi: 10.1042/CS20160010. 27798228

[32] Roman YM. The role of uric acid in human health: insights from the uricase gene. J Pers Med [Internet]. 2023;13(9):1409. doi: 10.3390/jpm13091409. 37763176 PMC10532990

[33] de Abreu MFS, Wegermann CA, Ceroullo MS, et al. Ten years milestones in xanthine oxidase inhibitors discovery: febuxostat-based inhibitors trends, bifunctional derivatives, and automatized screening assays. Organics [Internet]. 2022;3(4):380–414. doi: 10.3390/org3040026.

[34] Maia LB, Pereira V, Mira L, et al. Nitrite reductase activity of rat and human xanthine oxidase, xanthine dehydrogenase, and aldehyde oxidase: evaluation of their contribution to NO formation in vivo. Biochemistry. 2015;54(3):685–710. doi: 10.1021/bi500987w. 25537183

[35] Agarwal A, Banerjee A, Banerjee UC. Xanthine oxidoreductase: a journey from purine metabolism to cardiovascular excitation-contraction coupling. Crit Rev Biotechnol. 2011;31(3):264–280. doi: 10.3109/07388551.2010.527823. 21774633

[36] Nishino T, Okamoto K. The role of the [2Fe–2S] cluster centers in xanthine oxidoreductase. J Inorg Biochem. 2000;82(1–4):43–49. doi: 10.1016/s0162-0134(00)00165-3. 11132637

[37] Harris CM, Sanders SA, Massey V. Role of the flavin midpoint potential and NAD binding in determining NAD versus oxygen reactivity of xanthine oxidoreductase. J Biol Chem. 1999;274(8):4561–4569. doi: 10.1074/jbc.274.8.4561. 9988690

[38] Ribeiro PMG, Fernandes HS, Maia LB, et al. The complete catalytic mechanism of xanthine oxidase: a computational study. Inorg Chem Front. 2021;8(2):405–416. doi: 10.1039/D0QI01029D.

[39] Ichida K, Amaya Y, Okamoto K, et al. Mutations associated with functional disorder of xanthine oxidoreductase and hereditary xanthinuria in humans. Int J Mol Sci [Internet]. 2012;13(11):15475–15495. doi: 10.3390/ijms131115475. 23203137 PMC3509653

[40] Cerqueira N, Pakhira B, Sarkar S. Theoretical studies on mechanisms of some Mo enzymes. J Biol Inorg Chem. 2015;20(2):323–335. doi: 10.1007/s00775-015-1237-7. 25698503

[41] Unno T, Sugimoto A, Kakuda T. Scavenging effect of tea catechins and their epimers on superoxide anion radicals generated by a hypoxanthine and xanthine oxidase system. J Sci Food Agric. 2000;80(5):601–606. doi: 10.1002/(SICI)1097-0010(200004)80:5<601::AID-JSFA581>3.0.CO;2-O.

[42] Hille R, Hall J, Basu P. The mononuclear molybdenum enzymes. Chem Rev. 2014;114(7):3963–4038. doi: 10.1021/cr400443z. 24467397 PMC4080432

[43] Berry CE, Hare JM. Xanthine oxidoreductase and cardiovascular disease: molecular mechanisms and pathophysiological implications. J Physiol. 2004;555(Pt 3):589–606. doi: 10.1113/jphysiol.2003.055913. 14694147 PMC1664875

[44] Harrison R. Structure and function of xanthine oxidoreductase: where are we now? Free Radic Biol Med. 2002;33(6):774–797. doi: 10.1016/s0891-5849(02)00956-5. 12208366

[45] Srinivasan S, Kalaiselvi P, Sakthivel R, et al. Uric acid: an abettor or protector in calcium oxalate urolithiasis? Biochemical study in stone formers. Clin Chim Acta. 2005;353(1–2):45–51. doi: 10.1016/j.cccn.2004.09.024. 15698589

[46] Li L, Zhang Y, Zeng C. Update on the epidemiology, genetics, and therapeutic options of hyperuricemia. Am J Transl Res. 2020;12(7):3167–3181. 32774692 PMC7407685

[47] Vadakedath S, Kandi V. Probable potential role of urate transporter genes in the development of metabolic disorders. Cureus. 2018;10(3):e2382. doi: 10.7759/cureus.2382. 29850377 PMC5973493

[48] Jin M, Yang F, Yang I, et al. Uric acid, hyperuricemia and vascular diseases. Front Biosci (Landmark Ed). 2012;17(2):656–669. doi: 10.2741/3950. 22201767 PMC3247913

[49] Ridker PM, Wilson PWF, Grundy SM. Should C-reactive protein be added to metabolic syndrome and to assessment of global cardiovascular risk? Circulation. 2004;109(23):2818–2825. doi: 10.1161/01.CIR.0000132467.45278.59. 15197153

[50] Ravindran S, Munusamy S. Renoprotective mechanisms of sodium-glucose co-transporter 2 (SGLT2) inhibitors against the progression of diabetic kidney disease. J Cell Physiol. 2022;237(2):1182–1205. doi: 10.1002/jcp.30621. 34713897

[51] Sekine M, Okamoto K, Pai EF, et al. Allopurinol and oxypurinol differ in their strength and mechanisms of inhibition of xanthine oxidoreductase. J Biol Chem. 2023;299(9):105189. doi: 10.1016/j.jbc.2023.105189. 37625592 PMC10511816

[52] Torres RJ, Prior C, Puig JG. Efficacy and safety of allopurinol in patients with hypoxanthine-guanine phosphoribosyltransferase deficiency. Metabolism. 2007;56(9):1179–1186. doi: 10.1016/j.metabol.2007.04.013. 17697859

[53] Maghsoud Y, Dong C, Cisneros GA. Investigation of the inhibition mechanism of xanthine oxidoreductase by oxipurinol: a computational study. J Chem Inf Model. 2023;63(13):4190–4206. doi: 10.1021/acs.jcim.3c00624. 37319436 PMC10405278

[54] Pál P, Nivorozhkin A, Csaba S. Therapeutic effects of xanthine oxidase inhibitors: renaissance half a century after the discovery of allopurinol. Pharmacol Rev. 2006;58(1):87. 16507884 10.1124/pr.58.1.6 PMC2233605

[55] Stocker SL, Williams KM, McLachlan AJ, et al. Pharmacokinetic and pharmacodynamic interaction between allopurinol and probenecid in healthy subjects. Clin Pharmacokinet. 2008;47(2):111–118. doi: 10.2165/00003088-200847020-00004. 18193917

[56] Day RO, Graham GG, Hicks M, et al. Clinical pharmacokinetics and pharmacodynamics of allopurinol and oxypurinol. Clin Pharmacokinet. 2007;46(8):623–644. doi: 10.2165/00003088-200746080-00001. 17655371

[57] Kumar R, Joshi G, Kler H, et al. Toward an understanding of structural insights of xanthine and aldehyde oxidases: an overview of their inhibitors and role in various diseases. Med Res Rev. 2018;38(4):1073–1125. doi: 10.1002/med.21457. 28672082

[58] Cantor JR, Abu-Remaileh M, Kanarek N, et al. Physiologic medium rewires cellular metabolism and reveals uric acid as an endogenous inhibitor of UMP synthase. Cell. 2017;169(2):258–272. e17. doi: 10.1016/j.cell.2017.03.023. 28388410 PMC5421364

[59] Kamatani N, Jinnah HA, Hennekam RCM, et al. 6—Purine and pyrimidine metabolism. In: Pyeritz RE, Korf BR, Grody WW, editors. Emery and rimoin’s principles and practice of medical genetics and genomics. 7th ed. London, United Kingdom: Academic Press; 2021. p. 183–234.

[60] Sugamura K, Keaney JF. Reactive oxygen species in cardiovascular disease. Free Radic Biol Med. 2011;51(5):978–992. doi: 10.1016/j.freeradbiomed.2011.05.004. 21627987 PMC3156326

[61] van der Pol KH, Wever KE, Verbakel M, et al. Allopurinol to reduce cardiovascular morbidity and mortality: a systematic review and meta-analysis. PLoS One. 2021;16(12):e0260844. doi: 10.1371/journal.pone.0260844. 34855873 PMC8638940

[62] Gois PHF, Souza ERM. Pharmacotherapy for hyperuricaemia in hypertensive patients. Cochrane Database Syst Rev. 2020;9(9):Cd008652. doi: 10.1002/14651858.CD008652.pub4. 32877573 PMC8094453

[63] Badve SV, Pascoe EM, Tiku A, et al. Effects of allopurinol on the progression of chronic kidney disease. N Engl J Med. 2020;382(26):2504–2513. doi: 10.1056/NEJMoa1915833. 32579811

[64] Mackenzie IS, Hawkey CJ, Ford I, et al. Allopurinol versus usual care in UK patients with ischaemic heart disease (ALL-HEART): a multicentre, prospective, randomised, open-label, blinded-endpoint trial. Lancet. 2022;400(10359):1195–1205. doi: 10.1016/S0140-6736(22)01657-9. 36216006

[65] ElShagea HN, ElKasabgy NA, Fahmy RH, et al. Freeze-Dried Self-Nanoemulsifying Self-Nanosuspension (SNESNS): a new approach for the preparation of a highly drug-loaded dosage form. AAPS PharmSciTech. 2019;20(7):258. doi: 10.1208/s12249-019-1472-2. 31332638

[66] Mukoyoshi M, Nishimura S, Hoshide S, et al. In vitro drug–drug interaction studies with febuxostat, a novel non-purine selective inhibitor of xanthine oxidase: plasma protein binding, identification of metabolic enzymes and cytochrome P450 inhibition. Xenobiotica. 2008;38(5):496–510. doi: 10.1080/00498250801956350. 18421623

[67] Love BL, Barrons R, Veverka A, et al. Urate-Lowering therapy for gout: focus on febuxostat. Pharmacotherapy: J Hum Pharmacol Drug Ther. 2010;30(6):594–608. doi: 10.1592/phco.30.6.594. 20500048

[68] Kamel B, Graham GG, Stocker SL, et al. A pharmacokinetic-pharmacodynamic study of a single dose of febuxostat in healthy subjects. Br J Clin Pharmacol. 2020;86(12):2486–2496. doi: 10.1111/bcp.14357. 32386239 PMC7688545

[69] Ernst ME, Fravel MA. Febuxostat: a selective xanthine-oxidase/xanthine-dehydrogenase inhibitor for the management of hyperuricemia in adults with gout. Clin Ther. 2009;31(11):2503–2518. doi: 10.1016/j.clinthera.2009.11.033. 20109996

[70] Strilchuk L, Fogacci F, Cicero AF. Safety and tolerability of available urate-lowering drugs: a critical review. Expert Opin Drug Saf. 2019;18(4):261–271. doi: 10.1080/14740338.2019.1594771. 30915866

[71] O’Dell JR, Brophy MT, Pillinger MH, et al. Comparative effectiveness of allopurinol and febuxostat in gout management. NEJM Evid. 2022;1(3):EVIDoa2100028. doi: 10.1056/evidoa2100028. PMC9012032 35434725

[72] Zhang F, Liu Z, Jiang L, et al. A randomized, double-Blind, non-Inferiority study of febuxostat versus allopurinol in hyperuricemic Chinese subjects with or without gout. Rheumatol Ther. 2019;6(4):543–557. doi: 10.1007/s40744-019-00173-8. 31531831 PMC6858416

[73] Peng Y-L, Tain Y-L, Lee C-T, et al. Comparison of uric acid reduction and renal outcomes of febuxostat vs allopurinol in patients with chronic kidney disease. Sci Rep. 2020;10(1):10734. doi: 10.1038/s41598-020-67026-1. 32612180 PMC7329906

[74] Pawar A, Desai RJ, Liu J, et al. Updated assessment of cardiovascular risk in older patients with gout initiating febuxostat versus allopurinol. J Am Heart Assoc. 2021;10(7):e020045. doi: 10.1161/JAHA.120.020045. 33764153 PMC8174329

[75] Kim H, Baek CH, Chang JW, et al. Febuxostat, a novel inhibitor of xanthine oxidase, reduces ER stress through upregulation of SIRT1-AMPK-HO-1/thioredoxin expression. Clin Exp Nephrol. 2020;24(3):205–215. doi: 10.1007/s10157-019-01804-8. 31677062

[76] Nomura J, Kobayashi T, So A, et al. Febuxostat, a xanthine oxidoreductase inhibitor, decreases NLRP3-dependent inflammation in macrophages by activating the purine salvage pathway and restoring cellular bioenergetics. Sci Rep. 2019;9(1):17314. doi: 10.1038/s41598-019-53965-x. 31754153 PMC6872548

[77] Amirshahrokhi K. Febuxostat attenuates ulcerative colitis by the inhibition of NF-κB, proinflammatory cytokines, and oxidative stress in mice. Int Immunopharmacol. 2019;76:105884. doi: 10.1016/j.intimp.2019.105884. 31499267

[78] Odake K, Tsujii M, Iino T, et al. Febuxostat treatment attenuates oxidative stress and inflammation due to ischemia-reperfusion injury through the necrotic pathway in skin flap of animal model. Free Radic Biol Med. 2021;177:238–246. doi: 10.1016/j.freeradbiomed.2021.10.033. 34737143

[79] Ashtar M, Tenshin H, Teramachi J, et al. The roles of ROS generation in RANKL-Induced osteoclastogenesis: suppressive effects of febuxostat. Cancers [Internet]. 2020;12(4):929. doi: 10.3390/cancers12040929. 32283857 PMC7226249

[80] Higa Y, Hiasa M, Tenshin H, et al. The xanthine oxidase inhibitor febuxostat suppresses adipogenesis and activates Nrf2. Antioxidants [Internet]. 2023;12(1):133. doi: 10.3390/antiox12010133. 36670994 PMC9854541

[81] Abdel-Wahab BA, El-Shoura EAM, Shafiuddin Habeeb M, et al. Febuxostat alleviates arsenic Trioxide-Induced renal injury in rats: insights on the crosstalk between NLRP3/TLR4, sirt-1/NF-κB/TGF-β signaling pathways, and miR-23b-3p, miR-181a-5b expression. Biochem Pharmacol. 2023;216:115794. doi: 10.1016/j.bcp.2023.115794. 37689273

[82] Zhang C, Tang L, Zhang Y, et al. Febuxostat, a xanthine oxidase inhibitor, regulated long noncoding RNAs and protected the brain after intracerebral hemorrhage. NeuroReport. 2023;34(14):703–712. doi: 10.1097/WNR.0000000000001945. 37556585

[83] Yan W, Zhang Y, Hu L, et al. Febuxostat inhibits MPP+-induced inflammatory response through inhibiting the JNK/NF-κB pathway in astrocytes. Neurotox Res. 2021;39(3):566–574. doi: 10.1007/s12640-020-00316-8. 33443645

[84] Lanaspa MA, Andres-Hernando A, Kuwabara M. Uric acid and hypertension. Hypertens Res. 2020;43(8):832–834. doi: 10.1038/s41440-020-0481-6. 32514150 PMC10000019

[85] Kuwabara M, Niwa K, Nishi Y, et al. Relationship between serum uric acid levels and hypertension among Japanese individuals not treated for hyperuricemia and hypertension. Hypertens Res. 2014;37(8):785–789. doi: 10.1038/hr.2014.75. 24671018

[86] Kuwabara M, Hisatome I, Niwa K, et al. Uric acid is a strong risk marker for developing hypertension from prehypertension. Hypertension. 2018;71(1):78–86. doi: 10.1161/HYPERTENSIONAHA.117.10370. 29203632 PMC5730471

[87] Bove M, Cicero AFG, Borghi C. The effect of xanthine oxidase inhibitors on blood pressure and renal function. Curr Hypertens Rep. 2017;19(12):95. doi: 10.1007/s11906-017-0793-3. 29071435

[88] Kuwabara M, Kanbay M, Hisatome I. Uric acid and hypertension because of arterial stiffness. Hypertension. 2018;72(3):582–584. doi: 10.1161/HYPERTENSIONAHA.118.11496. 29987106

[89] Dikalov SI, Ungvari Z. Role of mitochondrial oxidative stress in hypertension. Am J Physiol Heart Circ Physiol. 2013;305(10):H1417–H1427. doi: 10.1152/ajpheart.00089.2013. 24043248 PMC3840266

[90] Zhang B, Duan M, Long B, et al. Urate transport capacity of glucose transporter 9 and urate transporter 1 in cartilage chondrocytes. Mol Med Rep. 2019;20(2):1645–1654. 31257523 10.3892/mmr.2019.10426 PMC6625399

[91] Bhatnagar A. Environmental determinants of cardiovascular disease. Circ Res. 2017;121(2):162–180. doi: 10.1161/CIRCRESAHA.117.306458. 28684622 PMC5777598

[92] Lee T-S, Lu T-M, Chen C-H, et al. Hyperuricemia induces endothelial dysfunction and accelerates atherosclerosis by disturbing the asymmetric dimethylarginine/dimethylarginine dimethylaminotransferase 2 pathway. Redox Biol. 2021;46:102108. doi: 10.1016/j.redox.2021.102108. 34438260 PMC8390558

[93] Wang A, Tian X, Wu S, et al. Metabolic factors mediate the association between serum uric acid to serum creatinine ratio and cardiovascular disease. J Am Heart Assoc. 2021;10(23):e023054. doi: 10.1161/JAHA.121.023054. 34779219 PMC9075399

[94] Tian X, Zuo Y, Chen S, et al. Associations between changes in serum uric acid and the risk of myocardial infarction. Int J Cardiol. 2020;314:25–31. doi: 10.1016/j.ijcard.2020.03.083. 32333932

[95] Maloberti A, Mengozzi A, Russo E, et al. The results of the URRAH (Uric Acid Right for Heart Health) project: a focus on hyperuricemia in relation to cardiovascular and kidney disease and its role in metabolic dysregulation. High Blood Press Cardiovasc Prev. 2023;30(5):411–425. doi: 10.1007/s40292-023-00602-4. 37792253 PMC10600296

[96] Maloberti A, Giannattasio C, Bombelli M, et al. Hyperuricemia and risk of cardiovascular outcomes: the experience of the URRAH (Uric Acid Right for Heart Health) project. High Blood Press Cardiovasc Prev. 2020;27(2):121–128. doi: 10.1007/s40292-020-00368-z. 32157643

[97] Russo E, Viazzi F, Pontremoli R, et al. Association of uric acid with kidney function and albuminuria: the Uric Acid Right for heArt Hhealth (URRAH) project. J Nephrol. 2022;35(1):211–221. doi: 10.1007/s40620-021-00985-4. 33755930 PMC8803667

[98] Casiglia E, Tikhonoff V, Virdis A, et al. Serum uric acid/serum creatinine ratio as a predictor of cardiovascular events. Detection of prognostic cardiovascular cut-off values. J Hypertens. 2023;41(1):180–186. doi: 10.1097/HJH.0000000000003319. 36453660 PMC9794153

[99] Scheepers LEJM, Wei F-F, Stolarz-Skrzypek K, et al. Xanthine oxidase gene variants and their association with blood pressure and incident hypertension: a population study. J Hypertens. 2016;34(11):2147–2154. doi: 10.1097/HJH.0000000000001077. 27607461

[100] Miah R, Fariha KA, Sony SA, et al. Association of serum xanthine oxidase levels with hypertension: a study on Bangladeshi adults. Sci Rep. 2022;12(1):21727. doi: 10.1038/s41598-022-26341-5. 36526797 PMC9758161

[101] Viel EC, Benkirane K, Javeshghani D, et al. Xanthine oxidase and mitochondria contribute to vascular superoxide anion generation in DOCA-salt hypertensive rats. Am J Physiol Heart Circ Physiol. 2008;295(1):H281–H288. doi: 10.1152/ajpheart.00304.2008. 18487445 PMC2494748

[102] Boban M, Kocic G, Radenkovic S, et al. Circulating purine compounds, uric acid, and xanthine oxidase/dehydrogenase relationship in essential hypertension and end stage renal disease. Ren Fail. 2014;36(4):613–618. doi: 10.3109/0886022X.2014.882240. 24502620

[103] Jankov RP, Kantores C, Pan J, et al. Contribution of xanthine oxidase-derived superoxide to chronic hypoxic pulmonary hypertension in neonatal rats. Am J Physiol Lung Cell Mol Physiol. 2008;294(2):L233–L245. doi: 10.1152/ajplung.00166.2007. 18083771

[104] Furuhashi M, Higashiura Y, Koyama M, et al. Independent association of plasma xanthine oxidoreductase activity with hypertension in nondiabetic subjects not using medication. Hypertens Res. 2021;44(9):1213–1220. doi: 10.1038/s41440-021-00679-1. 34117403

[105] Kurajoh M, Fukumoto S, Yoshida S, et al. Uric acid shown to contribute to increased oxidative stress level independent of xanthine oxidoreductase activity in MedCity21 health examination registry. Sci Rep. 2021;11(1):7378. doi: 10.1038/s41598-021-86962-0. 33795813 PMC8016900

[106] Muraya N, Kadowaki D, Miyamura S, et al. Benzbromarone attenuates oxidative stress in angiotensin II- and Salt-Induced hypertensive model rats. Oxid Med Cell Longev. 2018;2018:7635274–7635278. doi: 10.1155/2018/7635274. 29967665 PMC6008799

[107] Yun L, Yu X, Xu R. Uric acid/superoxide dismutase can predict progression of gestational hypertension to preeclampsia. Front Cardiovasc Med. 2023;10:1148376. doi: 10.3389/fcvm.2023.1148376. 37063971 PMC10097916

[108] Trapé AA, Jacomini AM, Muniz JJ, et al. The relationship between training status, blood pressure and uric acid in adults and elderly. BMC Cardiovasc Disord. 2013;13(1):44. doi: 10.1186/1471-2261-13-44. 23799981 PMC3695764

[109] Tsukimori K, Yoshitomi T, Morokuma S, et al. Serum uric acid levels correlate with plasma hydrogen peroxide and protein carbonyl levels in preeclampsia. Am J Hypertens. 2008;21(12):1343–1346. doi: 10.1038/ajh.2008.289. 18802427

[110] Zhang J, Zheng R, Li H, et al. Serum uric acid and incident atrial fibrillation: a systematic review and dose–response meta-analysis. Clin Exp Pharmacol Physiol. 2020;47(11):1774–1782. doi: 10.1111/1440-1681.13374. 32621546

[111] Kawasoe S, Kubozono T, Yoshifuku S, et al. Uric acid level and prevalence of atrial fibrillation in a Japanese general population of 285,882. Circ J. 2016;80(12):2453–2459. doi: 10.1253/circj.CJ-16-0766. 27818462

[112] Ding M, Viet NN, Gigante B, et al. Elevated uric acid is associated with new‐onset atrial fibrillation: results from the Swedish AMORIS cohort. J Am Heart Assoc. 2023;12(3):e027089. doi: 10.1161/JAHA.122.027089. 36633024 PMC9973652

[113] Deng Y, Liu F, Yang X, et al. The key role of uric acid in oxidative stress, inflammation, fibrosis, apoptosis, and immunity in the pathogenesis of atrial fibrillation. Front Cardiovasc Med. 2021;8:641136. doi: 10.3389/fcvm.2021.641136. 33718459 PMC7952317

[114] Perez-Ruiz F, Dalbeth N, Bardin T. A review of uric acid, crystal deposition disease, and gout. Adv Ther. 2015;32(1):31–41. doi: 10.1007/s12325-014-0175-z. 25533440 PMC4311063

[115] Johnson RJ, Sanchez Lozada LG, Lanaspa MA, et al. Uric acid and chronic kidney disease: still more to do. Kidney Int Rep. 2023;8(2):229–239. doi: 10.1016/j.ekir.2022.11.016. 36815099 PMC9939362

[116] Singh JA, Cleveland JD. Comparative effectiveness of allopurinol versus febuxostat for preventing incident dementia in older adults: a propensity-matched analysis. Arthritis Res Ther. 2018;20(1):167. doi: 10.1186/s13075-018-1663-3. 30075731 PMC6091090

[117] Kushiyama A, Nakatsu Y, Matsunaga Y, et al. Role of uric acid metabolism-related inflammation in the pathogenesis of metabolic syndrome components such as atherosclerosis and nonalcoholic steatohepatitis. Mediators Inflamm. 2016;2016:8603115–8603164. doi: 10.1155/2016/8603164. PMC5192336 28070145

[118] Sandoo A, van Zanten JJ, Metsios GS, et al. The endothelium and its role in regulating vascular tone. Open Cardiovasc Med J. 2010;4(1):302–312. doi: 10.2174/1874192401004010302. 21339899 PMC3040999

[119] Grover-Páez F, Zavalza-Gómez AB. Endothelial dysfunction and cardiovascular risk factors. Diabetes Res Clin Pract. 2009;84(1):1–10. doi: 10.1016/j.diabres.2008.12.013. 19185380

[120] Chen C, Lü JM, Yao Q. Hyperuricemia-Related diseases and xanthine oxidoreductase (XOR) inhibitors: an overview. Med Sci Monit. 2016;22:2501–2512. doi: 10.12659/msm.899852. 27423335 PMC4961276

[121] Pallavi P, Harini K, Alshehri S, et al. From synthetic route of silica nanoparticles to theranostic applications. Processes. 2022;10(12):2595. doi: 10.3390/pr10122595.

[122] Bhoopathy J, Vedakumari Sathyaraj W, Yesudhason BV, et al. Haemostatic potency of sodium alginate/aloe vera/sericin composite scaffolds—preparation, characterisation, and evaluation. Artif Cells Nanomed Biotechnol. 2024;52(1):35–45. doi: 10.1080/21691401.2023.2293784. 38112317

[123] Girigoswami K, Pallavi P, Girigoswami A. Targeting cancer stem cells by nanoenabled drug delivery. In: Pathak S, Banerjee A, editors. Cancer stem cells: new horizons in cancer therapies. Singapore: Springer Singapore; 2020. p. 313–337.

[124] Vedakumari SW, Prabu P, Jancy SJV, et al. Radiopaque fibrin nanocomplex as a promising tool for X-ray ­imaging applications. Int J Biol Macromol. 2022;200:285–292. doi: 10.1016/j.ijbiomac.2021.12.164. 34995664

[125] Gowtham P, Girigoswami K, Pallavi P, et al. Alginate-Derivative encapsulated carbon coated Manganese-Ferrite nanodots for multimodal medical imaging. Pharmaceutics. 2022;14(12):2550. doi: 10.3390/pharmaceutics14122550. 36559045 PMC9782169

[126] Paramita P, Subramaniam VD, Murugesan R, et al. Evaluation of potential anti‐cancer activity of cationic liposomal nanoformulated lycopodium clavatum in colon cancer cells. IET Nanobiotechnol. 2018;12(6):727–732. doi: 10.1049/iet-nbt.2017.0106. 30104445 PMC8675948

[127] Jessy Mercy D, Girigoswami K, Girigoswami A. A mini review on biosensor advancements-emphasis on quantum dots. Results Chem. 2024;7:101271. doi: 10.1016/j.rechem.2023.101271.

[128] Bhatt S, Sharma JB, Kamboj R, et al. Design and optimization of febuxostat-loaded nano lipid carriers using full factorial design. Turk J Pharm Sci. 2021;18(1):61–67. doi: 10.4274/tjps.galenos.2019.32656. 33633486 PMC7957308

[129] Gurumukhi VC, Sonawane VP, Tapadiya GG, et al. Quality-by-design based fabrication of febuxostat-loaded nanoemulsion: statistical optimization, characterizations, permeability, and bioavailability studies. Heliyon. 2023;9(4):e15404. doi: 10.1016/j.heliyon.2023.e15404. 37128342 PMC10148101

[130] Al-Amodi YA, Hosny KM, Alharbi WS, et al. Investigating the potential of transmucosal delivery of febuxostat from oral lyophilized tablets loaded with a self-nanoemulsifying delivery system. Pharmaceutics [Internet]. 2020;12(6):534. doi: 10.3390/pharmaceutics12060534. 32531910 PMC7356236

[131] Singh S, Parashar P, Kanoujia J, et al. Transdermal potential and anti-gout efficacy of febuxostat from niosomal gel. J Drug Deliv Sci Technol. 2017;39:348–361. doi: 10.1016/j.jddst.2017.04.020.

[132] Patel B, Thakkar H. Formulation development of fast dissolving microneedles loaded with cubosomes of febuxostat: in vitro and in vivo evaluation. Pharmaceutics [Internet]. 2023;15(1):224. doi: 10.3390/pharmaceutics15010224. 36678853 PMC9863705

[133] Ali Z, Din FU, Zahid F, et al. Transdermal delivery of allopurinol-loaded nanostructured lipid carrier in the treatment of gout. BMC Pharmacol Toxicol. 2022;23(1):86. doi: 10.1186/s40360-022-00625-y. 36443818 PMC9703780

[134] Kandav G, Bhatt DC, Singh SK. Effect of different molecular weights of chitosan on formulation and evaluation of allopurinol-loaded nanoparticles for kidney targeting and in management of hyperuricemic nephrolithiasis. AAPS PharmSciTech. 2022;23(5):144. doi: 10.1208/s12249-022-02297-7. 35578122

[135] Kandav G, Bhatt DC, Jindal DK, et al. Formulation, optimization, and evaluation of allopurinol-loaded bovine serum albumin nanoparticles for targeting kidney in management of hyperuricemic nephrolithiasis. AAPS PharmSciTech. 2020;21(5):164. doi: 10.1208/s12249-020-01695-z. 32488630

[136] Abdulaal WH, Alhakamy NA, Hosny KM. Preparation and characterization of a thioctic acid nanostructured lipid carrier to enhance the absorption profile and limit the nephrotoxicity associated with allopurinol in the treatment of gout. J Drug Deliv Sci Technol. 2021;66:102859. doi: 10.1016/j.jddst.2021.102859.

[137] Ahuja BK, Jena SK, Paidi SK, et al. Formulation, optimization and in vitro–in vivo evaluation of febuxostat nanosuspension. Int J Pharm. 2015;478(2):540–552. doi: 10.1016/j.ijpharm.2014.12.003. 25490182

[138] Amin OM, Ammar A, Eladawy SA. Febuxostat loaded β-cyclodextrin based nanosponge tablet: an in vitro and in vivo evaluation. J Pharm Investig. 2020;50(4):399–411. doi: 10.1007/s40005-019-00464-w.

[139] Tayyab M, Haseeb MT, Alsahli TG, et al. Fabrication and optimization of febuxostat-loaded chitosan nanocarriers for better pharmacokinetics profile. Int J Biol Macromol. 2024;257(Pt 1):128448. doi: 10.1016/j.ijbiomac.2023.128448. 38042323

[140] Sun J, Du J, Liu X, et al. Preparation of chitosan-coated hollow tin dioxide nanoparticles and their application in improving the oral bioavailability of febuxostat. Int J Pharm: X. 2023;6:100199. 37521247 10.1016/j.ijpx.2023.100199 PMC10384222

[141] Aganyants HA, Nikohosyan G, Danielyan KE. Albumin microparticles as the carriers for allopurinol and applicable for the treatment of ischemic stroke. Int Nano Lett. 2016;6(1):35–40. doi: 10.1007/s40089-015-0169-0.

[142] Ye L, Gao Z, Rohani S. Intervertebral disk regeneration in a rat model by allopurinol–loaded chitosan/alginate hydrogel. Biomol Biomed. 2023;23(4):661–670. doi: 10.17305/bb.2022.8550. 36786280 PMC10351085

[143] Sharma N, Kumar S, Joshi G, et al. Formulation and characterization of febuxostat loaded nanostructured lipid carriers (NLCs)-gel for topical treatment of gout. Recent Pat Nanotechnol. 2022;16(3):250–258. doi: 10.2174/1872210515666210415114118. 33858317

[144] El-Shenawy AA, Abdelhafez WA, Ismail A, et al. Formulation and characterization of nanosized ethosomal formulations of antigout model drug (febuxostat) prepared by cold method: in vitro/ex vivo and in vivo assessment. AAPS PharmSciTech. 2019;21(1):31. doi: 10.1208/s12249-019-1556-z. 31858305

[145] Battelli MG, Bortolotti M, Polito L, et al. The role of xanthine oxidoreductase and uric acid in metabolic syndrome. Biochim Biophys Acta Mol Basis Dis. 2018;1864(8):2557–2565. doi: 10.1016/j.bbadis.2018.05.003. 29733945

[146] Benn CL, Dua P, Gurrell R, et al. Physiology of hyperuricemia and urate-lowering treatments. Front Med. 2018;5:160. doi: 10.3389/fmed.2018.00160. PMC5990632 29904633

[147] Hyon KC, Lucia Cea S, Yuqing Z, et al. Antihypertensive drugs and risk of incident gout among patients with hypertension: population based case-control study. BMJ. 2012;344:d8190. 22240117 10.1136/bmj.d8190 PMC3257215

[148] Ganesan T, Muthudoss P, Voguri RS, et al. A new febuxostat-telmisartan drug-drug cocrystal for gout-hypertension combination therapy. J Pharm Sci. 2022;111(12):3318–3326. doi: 10.1016/j.xphs.2022.08.022. 36028135

